# Role of Ceramide from Glycosphingolipids and Its Metabolites in Immunological and Inflammatory Responses in Humans

**DOI:** 10.1155/2015/120748

**Published:** 2015-11-01

**Authors:** Kazuhisa Iwabuchi, Hitoshi Nakayama, Ami Oizumi, Yasushi Suga, Hideoki Ogawa, Kenji Takamori

**Affiliations:** ^1^Institute for Environmental and Gender-Specific Medicine, Juntendo University Graduate School of Medicine, 2-1-1 Tomioka Urayasu, Chiba 2790021, Japan; ^2^Infection Control Nursing, Juntendo University Graduate School of Health Care and Nursing, Chiba 2790023, Japan; ^3^Laboratory of Biochemistry, Juntendo University School of Health Care and Nursing, Chiba 2790023, Japan; ^4^Department of Dermatology, Juntendo University Urayasu Hospital, Chiba 2790021, Japan

## Abstract

Glycosphingolipids (GSLs) are composed of hydrophobic ceramide and hydrophilic sugar chains. GSLs cluster to form membrane microdomains (lipid rafts) on plasma membranes, along with several kinds of transducer molecules, including Src family kinases and small G proteins. However, GSL-mediated biological functions remain unclear. Lactosylceramide (LacCer, CDw17) is highly expressed on the plasma membranes of human phagocytes and mediates several immunological and inflammatory reactions, including phagocytosis, chemotaxis, and superoxide generation. LacCer forms membrane microdomains with the Src family tyrosine kinase Lyn and the G*α*i subunit of heterotrimeric G proteins. The very long fatty acids C24:0 and C24:1 are the main ceramide components of LacCer in neutrophil plasma membranes and are directly connected with the fatty acids of Lyn and G*α*i. These observations suggest that the very long fatty acid chains of ceramide are critical for GSL-mediated outside-in signaling. Sphingosine is another component of ceramide, with the hydrolysis of ceramide by ceramidase producing sphingosine and fatty acids. Sphingosine is phosphorylated by sphingosine kinase to sphingosine-1-phosphate, which is involved in a wide range of cellular functions, including growth, differentiation, survival, chemotaxis, angiogenesis, and embryogenesis, in various types of cells. This review describes the role of ceramide moiety of GSLs and its metabolites in immunological and inflammatory reactions in human.

## 1. Introduction

Biological membranes are mainly composed of phospholipids, sphingolipids, cholesterol, and membrane-associated proteins. These molecules are nonhomogeneously distributed in membranes and can rearrange, leading to the formation of membrane “domains” with highly differentiated molecular compositions and supramolecular architectures, which are stabilized by lateral interactions among the membrane components. Although glycosphingolipids (GSLs) were originally thought to be structural components of plasma membranes [1], several experiments suggested that GSLs are involved in the regulation of numerous cellular functions [2]. The membrane lipid bilayer is a stable structure, constituting a physical boundary between intra- and extracellular environments. GSLs are expressed on the surface of cellular membranes. Based on their physicochemical properties, especially their many hydroxyl and acetamide groups, which can act as hydrogen bond donors and acceptors, GSLs form clusters through cis interactions [2]. There is a general consensus on the roles played by the ceramide moiety of GSLs in promoting the formation and stabilization of membrane lipid domains. In addition, ceramide was also shown to be involved in GSL-mediated functions and several biological activities [3, 4]. Ceramide is composed of sphingosine and fatty acid chains. We recently showed that very long fatty acid chains of ceramide, such as C24:0 and C24:1, are responsible for the direct connection between lactosylceramide (LacCer, CDw17) and palmitoylated signal transducer molecules [5]. Moreover, the phosphorylated product of sphingosine, sphingosine-1-phosphate (S1P), was shown to be important in immunological, especially inflammatory reactions [4, 6].

This review describes the role of the fatty acid chains of ceramide in GSL-mediated outside-in signaling in promoting GSL-enriched domain-mediated cellular functions, as well as the activities of S1P in inflammatory reactions of keratinocytes in human.

## 2. Organization of GSL-Enriched Lipid Microdomains

GSLs on biological membranes tend to form specific domains with several types of molecules. The most studied GSL-enriched domains are membrane lipid microdomains, called lipid rafts, defined by their GSL- and cholesterol-rich nature, enrichment in GPI-anchored proteins and membrane-anchored signaling molecules, and cytoskeletal association [7, 8]. As shown in artificial membrane models, GSLs tend to form clusters [9], with this cluster formation confirmed in intact cells by immunoelectron microscopy [10–12]. The GSL-enriched microdomains on plasma membranes have a diameter of 50–100 nm and include signal transducer molecules, such as Src family kinases [11, 12]. GSLs that contain saturated fatty acid chains with higher transition temperatures [13] show ordered, less fluid, liquid phase. Cholesterol is composed of a highly hydrophobic sterol-ring system and 3-hydroxy moiety, the only hydrophilic part of the molecule. The small cholesterol sterol-ring system and the ceramide moiety of sphingolipids are thought to interact via hydrogen bonds and hydrophobic van der Waal's interactions [14]. In addition, hydrophilic interactions between sugar moieties of GSLs promote the lateral association of GSLs and cholesterol. In contrast, phospholipids have low acyl chain melting temperatures and unsaturated acyl chains. Phospholipids tend to be loosely packaged in bilayers, resulting in the formation of liquid-disordered membranes that allow rapid lateral and rotational movement of lipids [15]. These interactions result in the separation of GSL- and cholesterol-enriched lipid microdomains from other phospholipids in the cell membrane and the formation of distinct domains.

**Figure 1:**
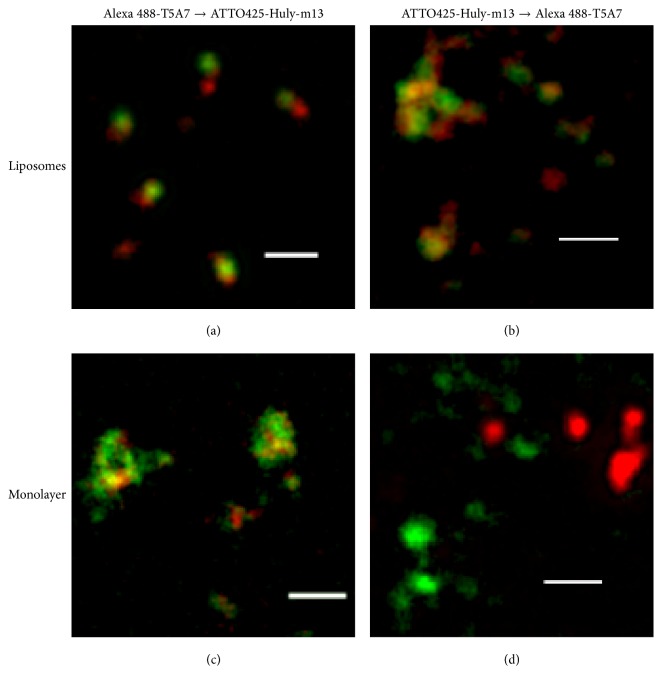
Stimulated emission depletion (STED) microscopic observation. The LacCer/DOPC liposomes (a, b) and LacCer and DOPC in ethanol (c, d) were coated onto the back surfaces of 96-well NUNC Immunoplates, followed by overnight incubation at room temperature with gentle shaking. The chemical condition of the backside surface of the plate was the same as the surfaces of the wells. The coated plates were blocked with BSA, sequentially stained with Alexa 488-T5A7 (green) ATTO425-Huly-m13 (red) (a, c) or ATTO425-conjugated Huly-m13 (red)→Alexa 488-conjugated T5A7 (green) (b, d), and viewed under a TCS STED CW superresolution microscope (Leica), with signals detected using a GaAsP hybrid detection system (Leica). Deconvolution was performed using Huygens STED deconvolution software (Leica). The panels on the right show enlargements of those on the left. White bars depict 1 *μ*m.

Electron microscopy using labeled anti-GSL antibodies has revealed GSL clusters on the surface of glycosphingolipid/phosphatidylcholine (PC) liposomes, even in the absence of sphingomyelin (SM) and cholesterol [2]. LacCer forms clusters, consisting of LacCer-enriched microdomains, on plasma membranes [11]. The anti-LacCer mAbs T5A7 and Huly-m13 recognized LacCer on human neutrophils, but only T5A7 recognized LacCer on mouse neutrophils. Interestingly, Huly-m13 but not T5A7 can be used for immunoprecipitation [16], suggesting a difference in binding and/or cluster formation of Huly-m13 and T5A7 to LacCer-enriched microdomains. Indeed, stimulated emission depletion (STED) superresolution microscopy showed that T5A7 and Huly-m13 bind to different regions of the same LacCer/dioleoylphosphatidylcholine (DOPC) liposomes (Figure 1) [17]. LacCer-enriched microdomains are composed of LacCer, SM, phospholipids, and cholesterol. Surface plasmon resonance analysis showed that reduction of the LacCer content in the DOPC/cholesterol/LacCer/SM lipid layer markedly decreased Huly-m13 but not T5A7 binding to LacCer [17], suggesting that the content of LacCer in LacCer-enriched microdomains affects the binding avidity of Huly-m13 to LacCer. In contrast, the molecular species of PC, including DOPC, dipalmitoylphosphatidylcholine (DPPC), and palmitoyl-oleoyl-phosphatidylcholine (POPC), did not affect the binding avidity of Huly-m13 to LacCer-coated plastic wells. Lactose inhibited the binding of Huly-m13 to LacCer/DOPC liposome-coated and DOPC/LacCer mixture-coated plastic wells, suggesting that Huly-m13 binds only to LacCer clusters in LacCer-enriched microdomains. In contrast, the binding avidity of T5A7 to LacCer-coated plastic wells was much weaker than its binding avidity to DOPC/LacCer-, POPC/LacCer-, and DPPC/LacCer mixture-coated wells, suggesting that the binding of T5A7 to LacCer is affected by PC. The ability of lactose to inhibit the binding of T5A7 to DOPC/LacCer liposome-coated plastic wells was similar to its ability to inhibit the binding of Huly-m13 [17]. In contrast, lactose inhibition of T5A7 binding to DOPC/LacCer mixture-coated plastic wells was significantly lower than its inhibition of Huly-m13 binding, suggesting that T5A7 recognizes the PC-enhanced three-dimensional structure of LacCer clusters. Thus, Huly-m13 may bind to the core region of lactose clusters in LacCer-enriched domains, while T5A7 binds to “dispersed” LacCer clusters in the phase boundary regions of these microdomains. These findings suggest that the specificities of these antibodies against the same GSLs are dependent on the organizations of the GSLs and molecules surrounding the GSL-enriched domains.

## 3. GSL Metabolism Diseases

Disorders of the degradation of GSLs sometimes cause human diseases [18, 19]. For degradation, GSLs are endocytosed and reach endosomes and other organelles. Then, those molecules are constitutively degraded by their suitable catabolic enzymes. When the activities of lysosomal enzymes are impaired, degradation is not able to proceed normally and undegraded molecules accumulate in the organelle and intracellular membranes, causing several metabolism diseases. For instance, genetic disorder of glucocerebrosidase (GBA) (EC 3.2.1.45; [20]), Gaucher disease, results in accumulation of GlcCer and its deacetylated form glucosylsphingosine is caused by abnormality of GBA. Gaucher disease is a multisystem disorder whose features include peripheral blood cytopenias, hepatosplenomegaly, bone disease, and neurological manifestations in some cases [21]. The form of intravenous enzyme replacement therapy in the 1990s has been developed and resulted in dramatic improvements in haematological and visceral disease [22]. Recognition of complications, including multiple myeloma and Parkinson disease, has challenged the traditional macrophage-centric view of the pathophysiology of this disorder. However, the pathways by which enzyme deficiency results in the clinical manifestations of this disorder remain obscure. In spinal cords of amyotrophic lateral sclerosis (ALS) patients, levels of GM1, GM3, LacCer, GlcCer, GalCer, and ceramide were significantly elevated [23]. Furthermore, glucocerebrosidase-1, glucocerebrosidase-2, hexosaminidase, galactosylceramidase, *α*-galactosidase, and *β*-galactosidase activities were also elevated in those patients. Inhibition of glucosylceramide synthesis accelerated disease course in ALS model mice, whereas infusion of exogenous GM3 significantly slowed the onset of paralysis and increased survival. These observations suggest that GSLs and their metabolism are likely important participants in pathogenesis of ALS. Further studies about GSL metabolism pathways in GSL-related disease will serve to advance our understanding of other associated disorders.

## 4. GSL- and Ceramide-Enriched Membrane Microdomains Are Binding Targets for Pathogenic Microorganisms

Over the last 30 years, many studies have indicated that GSLs expressed on the cell surface may act as binding sites for microorganisms. The binding avidities of microorganisms to several types of GSL [24–27] suggest that GSLs are involved in host-pathogen interactions. Indeed, microorganisms have been shown to recognize and enter host cells* via *GSL-enriched membrane microdomains on the cells [28]. Among GSLs, LacCer has been well described to bind to several kinds of microorganisms, including viruses and fungi [27]. For instance,* Candida albicans* specifically bind to LacCer though the binding of *β*-1,6-long glucosyl side-chain-branched *β*-1,3-glucan to LacCer-enriched domains [26, 29]. It is also well known that microorganisms-derived toxins, such as Shiga toxin, specifically bind to GSLs [30–32]. Furthermore, a sphingolipid metabolite, ceramide, has been demonstrated to play a crucial role in pulmonary infection and inflammation [33].Ceramide, which is degraded product of GSLs and sphingomyelin, has been reported to form ceramide-rich membrane platforms and involve uptake of several microorganisms including* Pseudomonas aeruginosa*. Abnormal amounts of enzymes involved in the synthesis of ceramide have been demonstrated in emphysematic smokers and in patients with severe sepsis [34]. Therefore, GSLs and their metabolites play important roles in infection and inflammation.

## 5. Fatty Acid Chains of Ceramide Are Indispensable for GSL-Mediated Signaling

GSLs have been reported to interact with membrane proteins and modulate the properties of these proteins [2, 25]. In addition, certain proteins, including glycosylphosphatidylinositol- (GPI-) anchored and palmitoylated proteins, tend to enter GSL-enriched membrane microdomains [13]. These observations suggested that GSLs may be involved in transferring information across membranes. However, the mechanism by which GSLs interact with proteins and mediate outside-in signaling is unclear. The ceramide moiety consists of a long chain base linked to a fatty acid chain. Sphingosine containing C18 carbons [(2*S*,3*R*,4*E*)-2-amino-1,3-dihydroxy-octadecene] is generally the main structure in mammals, but a structure containing 20 carbons is relatively abundant in neurons. However, the fatty acid content of GSL ceramide is highly heterogeneous [35]. Ceramide is synthesized by ceramide synthases (CerS) 1–6, each of which uses a restricted subset of fatty acyl-CoAs for N-acylation of the sphingoid long chain base [36]. The expression levels of genes encoding CerS are tissue specific, suggesting that the molecular varieties and expression patterns of GSLs are associated with the functions of these cells [37].

**Figure 2:**
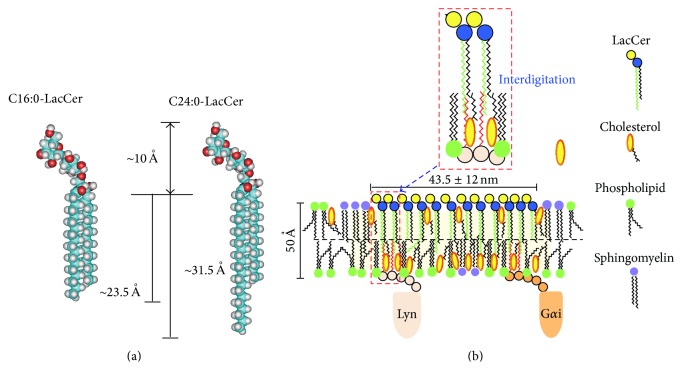
LacCer-enriched microdomains. (a) Size of LacCer microdomains containing C16:0 and C24:0 fatty acid chain. (b) LacCer forms lipid microdomains on plasma membrane of human neutrophils and acts as a signal transduction platform. The C24 fatty acid chains of LacCer interdigitate into inner leaflet of plasma membranes and directly interact with Lyn and G*α*i. These molecules associate with LacCer to mediate signaling from outside to inside, resulting in neutrophil chemotaxis, migration, and phagocytosis.

Although GSL-enriched microdomains have been implicated in a number of important membrane events [2, 38, 39], the molecular mechanisms responsible for GSL-mediated cell functions are still unclear. One of the main issues centers around the association of GSLs with signal transducer molecules localized on the cytosolic side. However, we recently analyzed LacCer-enriched microdomains in human neutrophilic lineage cells [38]. LacCer, along with the Src family kinase Lyn, forms lipid microdomains on the plasma membranes of human neutrophils and is involved in several cellular functions, including chemotaxis, phagocytosis, and superoxide generation, highly dependent on Lyn [16, 29, 38]. HL-60 cells differentiated into neutrophilic lineage cells by DMSO (D-HL-60) were found to acquire superoxide generating activity, but not through LacCer, despite their expression of LacCer on plasma membranes [38]. Most LacCer and Lyn were recovered in the microdomain fractions of neutrophils and D-HL-60 cells. Lipidomics analysis revealed that LacCer in the neutrophil plasma membrane was mainly composed of molecular species containing C16:0, C24:1, and C24:0 fatty acid chains, whereas over 70% of LacCer in the plasma membranes of D-HL-60 cells contained C16:0 fatty acid chains, but only about 14% were C24:1 and C24:0 [11]. Lyn was immunoprecipitated by anti-LacCer antibody in neutrophils but not D-HL-60 cells. Importantly, Lyn was coimmunoprecipitated by anti-LacCer antibody from the detergent resistant membrane (DRM) fraction of plasma membranes from C24:0 and C24:1, but not C16:0 or C22:0, LacCer-loaded D-HL-60 cells. Anti-LacCer antibody induced superoxide generation from D-HL-60 cells loaded with C24:0-LacCer, but not C16:0-LacCer. Lyn colocalized with LacCer-enriched domains of D-HL-60 cells loaded with C24:0-LacCer, but not C16:0-LacCer. These results suggested that the C24 fatty acid chain of LacCer is indispensable for connecting Lyn with LacCer-enriched microdomains. Knockdown of Lyn molecules by human Lyn-specific short interfering RNA (siRNA) in D-HL-60 cells completely abolished the effects of C24:1-LacCer loading function [11], suggesting that Lyn is crucial for C24-LacCer-mediated neutrophil function. Experiments using azide-photoactivatable tritium-labeled C24- and C16-LacCer revealed that C24- but not C16-LacCer directly associated with Lyn and a heterotrimeric G protein subunit G*α*i. These results confirm a specific direct interaction between C24-LacCer and the signal transduction molecules Lyn and G*α*i, which are associated with the cytoplasmic layer via palmitic acid chains (Figure 2). LacCer species with very long fatty acids are indispensable for Lyn-coupled LacCer-enriched membrane microdomain-mediated neutrophil functions.

GPI-anchored proteins are composed of glycerol phospholipids, which do not have C24 fatty acid chains, suggesting that GPI-anchored proteins are not able to form large clusters by themselves and cannot directly connect with signal transduction molecules through fatty acid chains. To mediate cell functions, GPI-anchored proteins require signal transduction molecule-coupled transmembrane proteins or GSL-enriched domains, such as LacCer-enriched domains [40]. Further studies are required to determine the organization and signaling mechanisms of membrane microdomains.

## 6. Role of Ceramide Metabolite Sphingosine-1-phosphate in Immunological Reactions of Human Keratinocytes

The epidermis consists of a single layer of proliferating undifferentiated keratinocytes, the stratum basale, and several superficial layers of the stratum spinosum and stratum granulosum (SG), which form the stratum corneum (SC). The SC acts as an air-liquid interface barrier to avoid drying of tissues in contact with air. Ceramide is the main component of SC and is important for the water retention and permeability barrier functions of SC. Ceramides account for 30–40% of SC lipids [41, 42]. All ceramide molecules in the SC are derived from GlcCer and SM [43]. CDase hydrolyzed ceramide to yield sphingosine and fatty acids. Sphingosine can be phosphorylated by sphingosine kinase to form S1P, a molecule involved in a wide range of cellular functions, including growth, differentiation, survival, chemotaxis, angiogenesis, and embryogenesis, in various types of cells [44, 45]. S1P was shown to inhibit keratinocyte proliferation, to promote corneocyte differentiation [46], and to chemoattract keratinocytes. Roles of S1P in skin immunological functions have been demonstrated in mouse models [45, 47–51]. Mice are the good experimental tool of choice for the majority of immunologists, and the study of immune responses in mice has provided considerable insight into human immune system function. However, there are significant differences in immunological reactions between mice and human [52]. Little is known, however, about the role of ceramide metabolites in the immunological functions of differentiating keratinocytes.

**Figure 3:**
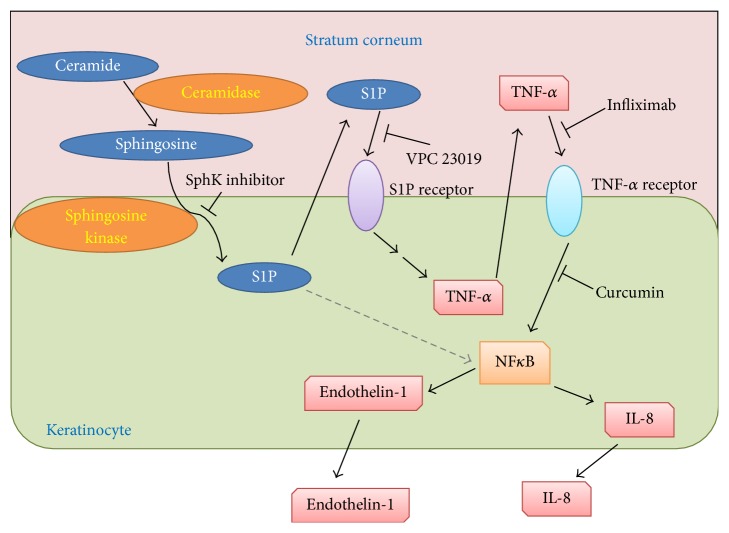
Schematic mechanism of the production of inflammatory mediators by ceramide metabolites in human keratinocytes. PaCDase degrades ceramide into sphingosine in the stratum corneum, and sphingosine is converted to S1P by SphK of keratinocytes. S1P is then released extracellularly and binds to S1P receptors, resulting in the production and release of TNF-*α*. The released TNF-*α* binds to TNF-*α* receptors, activating NF-*κ*B and inducing the production of IL-8 and endothelin-1.

A neutral CDase from* Pseudomonas aeruginosa *AN17 (PaCDase) isolated from a patient with atopic dermatitis (AD) was shown to require detergents to hydrolyze ceramide [53].* Staphylococcus aureus*-derived lipids, which consist primarily of cardiolipin and phosphatidylglycerol, enhanced the PaCDase hydrolysis of normal ceramide and of human skin-specific omega-hydroxyacyl ceramide in the absence of detergents [11]. A three-dimensionally cultured human primary keratinocyte (3D keratinocyte) culture system has been utilized to simulate epidermal differentiation at its air-liquid interface, resulting in the generation of basal, spinous, and granular layers and an SC, with the latter displaying permeability barrier functions [54]. Treatment of 3D keratinocytes with PaCDase and water-soluble stimulants of keratinocytes, including trypsin,* Dermatophagoides pteronyssinus* class 1 allergen (Der p1), and* Dermatophagoides farinae* allergen (Der f1) had no effect on the expression of any of the genes in our DNA microarray analysis [55], indicating that the SC of the 3D keratinocyte culture acts as a permeability barrier. Triton X-100 is a detergent that reduces permeability barrier functions, thereby moderately increasing transepidermal water loss and the production of erythema on human skin [56]. In the presence of 0.1% Triton X-100, PaCDase markedly enhanced TNF-*α* mRNA expression in 3D keratinocytes, an increase not observed in cells treated with Triton X-100 alone [55]. TNF-*α* mRNA expression was not enhanced by heat-inactivated or mutant PaCDase, suggesting that ceramide metabolites induce TNF-*α* mRNA expression in keratinocytes. TNF-*α*, a critical cytokine in several dermatological diseases [57], is secreted by keratinocytes [58] and shown to be involved in the progression of atopic dermatitis (AD) [59]. Among the metabolites of ceramide, only sphingosine and S1P enhanced TNF-*α* mRNA levels in 3D keratinocytes. S1P is synthesized from sphingosine by sphingosine kinase (SphK) and stimulates 3D keratinocytes through specific receptors [60]. Both the specific SphK inhibitor CAS 1177741-83-1 and the S1P receptor antagonist VPC 23019 suppressed the PaCDase-induced expression of TNF-*α* mRNA in 3D keratinocytes. S1P is generally considered to stimulate cells through plasma membrane G protein-coupled receptors, for example, S1P1–S1P5 [61]. S1P was recently shown to activate NF-*κ*B [62] independently of S1P receptors [63]. However, VPC2301, a competitive antagonist for S1P1 and S1P3 receptors, inhibited the PaCDase-enhanced gene expression not only of TNF-*α* but also of endothelin-1 and IL-8 [55]. Thus, the S1P-induced production of these inflammatory mediators is mediated by S1P receptors in human primary keratinocytes in a 3D culture system. cDNA microarray analysis showed that S1P strongly upregulated the expression of endothelin-1, CXCL1, TNF-*α*, *β*-defensin 5, IL-8, CXCL2, interferon regulatory factor 1, GADD45 gamma, and IL-23*α* subunit mRNAs [55]. IL-8, CXCL1, and CXCL2 have been reported to be upregulated in the lesional skin of patients with AD and psoriasis [64]. S1P also enhanced the expression of claudin-4 mRNA, which has been observed in more layers of psoriatic than normal epidermis [65]. TNF-*α* can induce the production of endothelin-1 and IL-8 by human keratinocytes [66, 67]. Epidermal keratinocytes produce and respond to TNF-*α* via TNFR1 [68]. Infliximab, a chimeric IgG1*κ* monoclonal antibody against human TNF-*α*, inhibits the TNF-*α*-mediated production of IL-8 by keratinocytes [69]. PaCDase-induced phosphorylation of NF-*κ*B p65 was markedly suppressed by infliximab [55]. The NF-*κ*B inhibitor curcumin inhibited PaCDase-induced expression of IL-8 and endothelin-1 mRNAs but not of TNF-*α* mRNA. TNF-*α* induces IL-8 production via NF-*κ*B [70]. Therefore, it is likely that S1P induces TNF-*α* production and release from 3D keratinocytes via S1P receptors, resulting in TNF-*α* induction of cytokine production through NF-*κ*B-mediated signal transduction (Figure 3). TNF-*α* is a critical cytokine in psoriatic immunopathology, and the development of an effective strategy is required to counteract its effects [57]. Infliximab, which is used to treat patients with plaque psoriasis, psoriatic arthritis, pustular psoriasis (excluding localized type), and psoriatic erythroderma [71, 72], downregulates antiapoptotic proteins in regressing psoriatic skin [72]. The effects of infliximab have also been evaluated in other inflammatory dermatoses and in systemic diseases involving the skin, pityriasis rubra pilaris, pyoderma gangrenosum, and cutaneous sarcoidosis [73]. AD is characterized by a marked reduction in ceramides in the SC of lesional and nonlesional forearms [74, 75] and by increased activities of the enzymes ceramidase (CDase). The metabolic conversion of ceramide to S1P has been found to protect keratinocytes against UVB-induced, ceramide-mediated apoptosis [76]. These observations suggest that ceramide metabolites, especially S1P, are involved in AD. AD is a common pruritic, inflammatory skin disorder [77]. Chronic, localized, or even generalized pruritus is the diagnostic hallmark of AD. Histamine H1-receptor blockers are used to treat all types of itch resulting from serious skin diseases, such as AD, as well as from renal and liver diseases. However, they often lack efficacy in chronic itch, a profound clinical problem that decreases quality of life [78]. Nerve density in the epidermis is partly involved in itch sensitization in pruritic skin diseases, such as AD [79]. Endothelin-1 has been shown to elicit itch in humans [80–82]. The molecular pathways that contribute to the transduction of itch responses to endothelin-1 do not require either PLC*β*_3_ or TRPV1 of neurons, which mediate histamine- and serotonin-induced itch responses, respectively [83]. Thus, keratinocyte-produced S1P may be involved in endothelin-1-mediated pruritus in AD. Therefore, atopic dermatitis may be exacerbated by treatment with S1P analogue FTY720.

## 7. Conclusion

Several reports have described the roles of ceramide metabolites in immunological and inflammatory diseases [84–88]. However, the physiological roles of GSL-enriched microdomains are largely undetermined, although much is known about the organization and functions of LacCer-enriched microdomains [24, 89, 90]. The analogous patterns of GSLs and motifs of PAMPs result in the generation of autoantibodies against these GSLs, inducing severe autoimmune inflammatory diseases [91]. Antibodies against neuronal tissues are involved in immune-mediated neurological disorders, with expression of several of these antibodies found to correlate with the pathophysiology of these diseases [92, 93]. Therefore, elucidation of their organization and structural specificities, based on interactions between GSLs and surrounding molecules, are important for understanding the physiological functions of GLS-enriched microdomains and their related diseases.

## Abbreviations

GSL: Glycosphingolipid
PaCDase: * Pseudomonas aeruginosa*-derived neutral ceramidase
S1P: Sphingosine-1-phosphate
3D keratinocytes: Three-dimensionally cultured human primary keratinocytes
SphK: Sphingosine kinase.

## References

[1] Ogretmen B., Hannun Y. A. (2004). Biologically active sphingolipids in cancer pathogenesis and treatment. *Nature Reviews Cancer*.

[2] Hakomori S. (2003). Structure, organization, and function of glycosphingolipids in membrane. *Current Opinion in Hematology*.

[3] Iwabuchi K., Nakayama H., Iwahara C., Takamori K. (2010). Significance of glycosphingolipid fatty acid chain length on membrane microdomain-mediated signal transduction. *FEBS Letters*.

[4] Snider A. J., Orr Gandy K. A., Obeid L. M. (2010). Sphingosine kinase: role in regulation of bioactive sphingolipid mediators in inflammation. *Biochimie*.

[5] Chiricozzi E., Ciampa M. G., Brasile G. (2015). Direct interaction, instrumental for signaling processes, between LacCer and Lyn in the lipid rafts of neutrophil-like cells. *Journal of Lipid Research*.

[6] Gonzalez-Cabrera P. J., Brown S., Studer S. M., Rosen H. (2014). S1P signaling: new therapies and opportunities. *F1000Prime Reports*.

[7] Simons K., Ikonen E. (1997). Functional rafts in cell membranes. *Nature*.

[8] Pike L. J. (2003). Lipid rafts: bringing order to chaos. *Journal of Lipid Research*.

[9] Prinetti A., Loberto N., Chigorno V., Sonnino S. (2009). Glycosphingolipid behaviour in complex membranes. *Biochimica et Biophysica Acta*.

[10] Murate M., Abe M., Kasahara K., Iwabuchi K., Umeda M., Kobayashi T. (2015). Transbilayer distribution of lipids at nano scale. *Journal of Cell Science*.

[11] Iwabuchi K., Prinetti A., Sonnino S. (2008). Involvement of very long fatty acid-containing lactosylceramide in lactosylceramide-mediated superoxide generation and migration in neutrophils. *Glycoconjugate Journal*.

[12] Fujita A., Cheng J., Fujimoto T. (2009). Segregation of GM1 and GM3 clusters in the cell membrane depends on the intact actin cytoskeleton. *Biochimica et Biophysica Acta: Molecular and Cell Biology of Lipids*.

[13] Sonnino S., Prinetti A., Mauri L., Chigorno V., Tettamanti G. (2006). Dynamic and structural properties of sphingolipids as driving forces for the formation of membrane domains. *Chemical Reviews*.

[14] Mukherjee S., Maxfield F. R. (2004). Membrane domains. *Annual Review of Cell and Developmental Biology*.

[15] Brown D. A., London E. (2000). Structure and function of sphingolipid- and cholesterol-rich membrane rafts. *Journal of Biological Chemistry*.

[16] Nakayama H., Yoshizaki F., Prinetti A. (2008). Lyn-coupled LacCer-enriched lipid rafts are required for CD11b/CD18-mediated neutrophil phagocytosis of nonopsonized microorganisms. *Journal of Leukocyte Biology*.

[17] Iwabuchi K., Masuda H., Kaga N. (2015). Properties and functions of lactosylceramide from mouse neutrophils. *Glycobiology*.

[18] Sandhoff K., Harzer K. (2013). Gangliosides and gangliosidoses: principles of molecular and metabolic pathogenesis. *The Journal of Neuroscience*.

[19] Platt F. M. (2014). Sphingolipid lysosomal storage disorders. *Nature*.

[20] Brady R. O., Gal A. E., Kanfer J. N., Bradley R. M. (1965). The metabolism of glucocerebrosides. 3. Purification and properties of a glucosyl- and galactosylceramide-cleaving enzyme from rat intestinal tissue. *The Journal of Biological Chemistry*.

[21] Cox T. M., Schofield J. P. (1997). Gaucher's disease: clinical features and natural history. *Bailliere's Clinical Haematology*.

[22] Thomas A. S., Mehta A., Hughes D. A. (2014). Gaucher disease: haematological presentations and complications. *British Journal of Haematology*.

[23] Dodge J. C., Treleaven C. M., Pacheco J. (2015). Glycosphingolipids are modulators of disease pathogenesis in amyotrophic lateral sclerosis. *Proceedings of the National Academy of Sciences of the United States of America*.

[24] Nakayama H., Ogawa H., Takamori K., Iwabuchi K. (2013). GSL-enriched membrane microdomains in innate immune responses. *Archivum Immunologiae et Therapiae Experimentalis*.

[25] Hakomori S.-I., Handa K., Iwabuchi K., Yamamura S., Prinetti A. (1998). New insights in glycosphingolipid function: ‘glycosignaling domain,’ a cell surface assembly of glycosphingolipids with signal transducer molecules, involved in cell adhesion coupled with signaling. *Glycobiology*.

[26] Jimenez-Lucho V., Ginsburg V., Krivan H. C. (1990). *Cryptococcus neoformans*, *Candida albicans,* and other fungi bind specifically to the glycosphingolipid lactosylceramide (GAl*β*1-4Glc*β*1-1Cer), a possible adhesion receptor for yeasts. *Infection and Immunity*.

[27] Karlsson K.-A. (1986). Animal glycolipids as attachment sites for microbes. *Chemistry and Physics of Lipids*.

[28] Mañes S., del Real G., Martínez-A C. (2003). Pathogens: raft hijackers. *Nature Reviews Immunology*.

[29] Sato T., Iwabuchi K., Nagaoka I. (2006). Induction of human neutrophil chemotaxis by *Candida albicans*-derived beta-1,6-long glycoside side-chain-branched beta-glucan. *Journal of Leukocyte Biology*.

[30] Zoja C., Buelli S., Morigi M. (2010). Shiga toxin-associated hemolytic uremic syndrome: pathophysiology of endothelial dysfunction. *Pediatric Nephrology*.

[31] Yamazaki Y., Horibata Y., Magatsuka Y., Hirabayashi Y., Hashikawa T. (2007). Fucoganglioside *α*-fucosyl(*α*-galactosyl)-GM1: a novel member of lipid membrane microdomain components involved in PC12 cell neuritogenesis. *Biochemical Journal*.

[32] Lingwood C. A. (1996). Role of verotoxin receptors in pathogenesis. *Trends in Microbiology*.

[33] Seitz A. P., Grassmé H., Edwards M. J., Pewzner-Jung Y., Gulbins E. (2015). Ceramide and sphingosine in pulmonary infections. *Biological Chemistry*.

[34] Esen M., Schreiner B., Jendrossek V. (2001). Mechanisms of *Staphylococcus aureus* induced apoptosis of human endothelial cells. *Apoptosis*.

[35] Kaga N., Kazuno S., Taka H., Iwabuchi K., Murayama K. (2005). Isolation and mass spectrometry characterization of molecular species of lactosylceramides using liquid chromatography-electrospray ion trap mass spectrometry. *Analytical Biochemistry*.

[36] Levy M., Futerman A. H. (2010). Mammalian ceramide synthases. *IUBMB Life*.

[37] Tidhar R., Ben-Dor S., Wang E., Kelly S., Merrill A. H., Futerman A. H. (2012). Acyl chain specificity of ceramide synthases is determined within a region of 150 residues in the tram-lag-CLN8 (TLC) domain. *The Journal of Biological Chemistry*.

[38] Iwabuchi K., Nagaoka I. (2002). Lactosylceramide-enriched glycosphingolipid signaling domain mediates superoxide generation from human neutrophils. *Blood*.

[39] Iwabuchi K., Yamamura S., Prinetti A., Handa K., Hakomori S.-I. (1998). GM3-enriched microdomain involved in cell adhesion and signal transduction through carbohydrate-carbohydrate interaction in mouse melanoma B16 cells. *Journal of Biological Chemistry*.

[40] Kusumi A., Shirai Y. M., Koyama-Honda I., Suzuki K. G. N., Fujiwara T. K. (2010). Hierarchical organization of the plasma membrane: investigations by single-molecule tracking vs. fluorescence correlation spectroscopy. *FEBS Letters*.

[41] Hamanaka S., Hara M., Nishio H., Otsuka F., Suzuki A., Uchida Y. (2002). Human epidermal glucosylceramides are major precursors of stratum corneum ceramides. *Journal of Investigative Dermatology*.

[42] Behne M., Uchida Y., Seki T., Ortiz De Montellano P., Elias P. M., Holleran W. M. (2000). Omega-hydroxyceramides are required for corneocyte lipid envelope (CLE) formation and normal epidermal permeability barrier function. *Journal of Investigative Dermatology*.

[43] Katoh M., Hamajima F., Ogasawara T., Hata K.-I. (2009). Assessment of human epidermal model LabCyte EPI-MODEL for in vitro skin irritation testing according to European Centre for the Validation of Alternative Methods (ECVAM)-validated protocol. *Journal of Toxicological Sciences*.

[44] Xia P., Wadham C. (2011). Sphingosine 1-phosphate, a key mediator of the cytokine network: juxtacrine signaling. *Cytokine and Growth Factor Reviews*.

[45] Herzinger T., Kleuser B., Schäfer-Korting M., Korting H. C. (2007). Sphingosine-1-phosphate signaling and the skin. *American Journal of Clinical Dermatology*.

[46] Vogler R., Sauer B., Kim D.-S., Schäfer-Korting M., Kleuser B. (2003). Sphingosine-1-phosphate and its potentially paradoxical effects on critical parameters of cutaneous wound healing. *Journal of Investigative Dermatology*.

[47] Arlt O., Schwiebs A., Japtok L. (2014). Sphingosine-1-phosphate modulates dendritic cell function: focus on non-migratory effects in vitro and in vivo. *Cellular Physiology and Biochemistry*.

[48] Schaper K., Dickhaut J., Japtok L. (2013). Sphingosine-1-phosphate exhibits anti-proliferative and anti-inflammatory effects in mouse models of psoriasis. *Journal of Dermatological Science*.

[49] Tsuji T., Yoshida Y., Iwatsuki R., Inoue M., Fujita T., Kohno T. (2012). Therapeutic approach to steroid-resistant dermatitis using novel immunomodulator FTY720 (Fingolimod) in combination with betamethasone ointment in NC/Nga mice. *Biological and Pharmaceutical Bulletin*.

[50] Bäumer W., Robach K., Mischke R. (2011). Decreased concentration and enhanced metabolism of sphingosine-1-phosphate in lesional skin of dogs with atopic dermatitis: disturbed sphingosine-1-phosphate homeostasis in atopic dermatitis. *Journal of Investigative Dermatology*.

[51] Reines I., Kietzmann M., Mischke R. (2009). Topical application of sphingosine-1-phosphate and FTY720 attenuate allergic contact dermatitis reaction through inhibition of dendritic cell migration. *Journal of Investigative Dermatology*.

[52] Mestas J., Hughes C. C. W. (2004). Of mice and not men: differences between mouse and human immunology. *The Journal of Immunology*.

[53] Voegeli T. S., Currie R. W. (2009). SiRNA knocks down Hsp27 and increases angiotensin II-induced phosphorylated NF-*κ*B p65 levels in aortic smooth muscle cells. *Inflammation Research*.

[54] Spöler F., Först M., Marquardt Y. (2006). High-resolution optical coherence tomography as a non-destructive monitoring tool for the engineering of skin equivalents. *Skin Research and Technology*.

[55] Oizumi A., Nakayama H., Okino N. (2014). Pseudomonas-derived ceramidase induces production of inflammatory mediators from human keratinocytes via sphingosine-1-phosphate. *PLoS ONE*.

[56] Kita K., Okino N., Ito M. (2000). Reverse hydrolysis reaction of a recombinant alkaline ceramidase of *Pseudomonas aeruginosa*. *Biochimica et Biophysica Acta*.

[57] Cho J.-W., Lee K.-S., Kim C.-W. (2007). Curcumin attenuates the expression of IL-1beta, IL-6, and TNF-alpha as well as cyclin E in TNF-alpha-treated HaCaT cells; NF-kappaB and MAPKs as potential upstream targets. *International Journal of Molecular Medicine*.

[58] Köck A., Schwarz T., Kirnbauer R. (1990). Human keratinocytes are a source for tumor necrosis factor *α*: evidence for synthesis and release upon stimulation with endotoxin or ultraviolet light. *The Journal of Experimental Medicine*.

[59] Boguniewicz M., Leung D. Y. M. (2011). Atopic dermatitis: a disease of altered skin barrier and immune dysregulation. *Immunological Reviews*.

[60] Lichte K., Rossi R., Danneberg K. (2008). Lysophospholipid receptor-mediated calcium signaling in human keratinocytes. *Journal of Investigative Dermatology*.

[61] Chi H. (2011). Sphingosine-1-phosphate and immune regulation: trafficking and beyond. *Trends in Pharmacological Sciences*.

[62] Siehler S., Wang Y., Fan X., Windh R. T., Manning D. R. (2001). Sphingosine 1-phosphate activates nuclear factor-*κ*B through Edg receptors. Activation through Edg-3 and Edg-5, but not Edg-1, in human embryonic kidney 293 cells. *Journal of Biological Chemistry*.

[63] Alvarez S. E., Harikumar K. B., Hait N. C. (2010). Sphingosine-1-phosphate is a missing cofactor for the E3 ubiquitin ligase TRAF2. *Nature*.

[64] Fujita H., Shemer A., Suárez-Fariñas M. (2011). Lesional dendritic cells in patients with chronic atopic dermatitis and psoriasis exhibit parallel ability to activate T-cell subsets. *The Journal of Allergy and Clinical Immunology*.

[65] Kirschner N., Poetzl C., von den Driesch P. (2009). Alteration of tight junction proteins is an early event in psoriasis: putative involvement of proinflammatory cytokines. *The American Journal of Pathology*.

[66] Ahn G. Y., Butt K. I., Jindo T., Yaguchi H., Tsuboi R., Ogawa H. (1998). The expression of endothelin-1 and its binding sites in mouse skin increased after ultraviolet B irradiation or local injection of tumor necrosis factor alpha. *Journal of Dermatology*.

[67] Lee C. S., Ko H. H., Seo S. J. (2009). Diarylheptanoid hirsutenone prevents tumor necrosis factor-alpha-stimulated production of inflammatory mediators in human keratinocytes through NF-kappaB inhibition. *International Immunopharmacology*.

[68] Lisby S., Faurschou A., Gniadecki R. (2007). The autocrine TNFalpha signalling loop in keratinocytes requires atypical PKC species and NF-kappaB activation but is independent of cholesterol-enriched membrane microdomains. *Biochemical Pharmacology*.

[69] Faurschou A., Gniadecki R., Wulf H. C. (2008). Infliximab inhibits DNA repair in ultraviolet B-irradiated premalignant keratinocytes. *Experimental Dermatology*.

[70] Barker J. N. W. N., Jones M. L., Mitra R. S. (1991). Modulation of keratinocyte-derived interleukin-8 which is chemotactic for neutrophils and T lymphocytes. *The American Journal of Pathology*.

[71] Torii H., Nakagawa H. (2011). Long-term study of infliximab in Japanese patients with plaque psoriasis, psoriatic arthritis, pustular psoriasis and psoriatic erythroderma. *Journal of Dermatology*.

[72] Kokolakis G., Giannikaki E., Stathopoulos E., Avramidis G., Tosca A. D., Krüger-Krasagakis S. (2012). Infliximab restores the balance between pro- and anti-apoptotic proteins in regressing psoriatic lesions. *British Journal of Dermatology*.

[73] Rigopoulos D., Korfitis C., Gregoriou S., Katsambas A. D. (2008). Infliximab in dermatological treatment: beyond psoriasis. *Expert Opinion on Biological Therapy*.

[74] Imokawa G., Abe A., Jin K., Higaki Y., Kawashima M., Hidano A. (1991). Decreased level of ceramides in stratum corneum of atopic dermatitis: an etiologic factor in atopic dry skin?. *Journal of Investigative Dermatology*.

[75] Spiekstra S. W., Dos Santos G. G., Scheper R. J., Gibbs S. (2009). Potential method to determine irritant potency in vitro—comparison of two reconstructed epidermal culture models with different barrier competency. *Toxicology in Vitro*.

[76] Moonen R. M. J., Reyes I., Cavallaro G., González-Luis G., Bakker J. A., Villamor E. (2010). The T1405N carbamoyl phosphate synthetase polymorphism does not affect plasma arginine concentrations in preterm infants. *PLoS ONE*.

[77] Ghazvini P., Pagan L. C., Rutledge T. K., Goodman H. S. (2010). Atopic dermatitis. *Journal of Pharmacy Practice*.

[78] Tominaga M., Takamori K. (2013). An update on peripheral mechanisms and treatments of itch. *Biological and Pharmaceutical Bulletin*.

[79] Tominaga M., Takamori K. (2014). Itch and nerve fibers with special reference to atopic dermatitis: therapeutic implications. *Journal of Dermatology*.

[80] Ferreira S. H., Romitelli M., de Nucci G. (1989). Endothelin-1 participation in overt and inflammatory pain. *Journal of Cardiovascular Pharmacology*.

[81] Katugampola R., Church M. K., Clough G. F. (2000). The neurogenic vasodilator response to endothelin-1: a study in human skin *in vivo*. *Experimental Physiology*.

[82] Kido-Nakahara M., Buddenkotte J., Kempkes C. (2014). Neural peptidase endothelin-converting enzyme 1 regulates endothelin 1-induced pruritus. *Journal of Clinical Investigation*.

[83] Imamachi N., Goon H. P., Lee H. (2009). TRPV1-expressing primary afferents generate behavioral responses to pruritogens via multiple mechanisms. *Proceedings of the National Academy of Sciences of the United States of America*.

[84] Yamaji T., Hanada K. (2015). Sphingolipid metabolism and interorganellar transport: localization of sphingolipid enzymes and lipid transfer proteins. *Traffic*.

[85] Tang X., Benesch M. G., Brindley D. N. (2015). Lipid phosphate phosphatases and their roles in mammalian physiology and pathology. *Journal of Lipid Research*.

[86] Rivera I. G., Ordonez M., Presa N. (2015). Sphingomyelinase D/ceramide 1-phosphate in cell survival and inflammation. *Toxins*.

[87] Nakamura H., Murayama T. (2014). The role of sphingolipids in arachidonic acid metabolism. *Journal of Pharmacological Sciences*.

[88] MacEyka M., Spiegel S. (2014). Sphingolipid metabolites in inflammatory disease. *Nature*.

[89] Iwabuchi K. (2015). Involvement of glycosphingolipid-enriched lipid rafts in inflammatory responses. *Frontiers in Bioscience*.

[90] Ekyalongo R. C., Nakayama H., Kina K., Kaga N., Iwabuchi K. (2015). Organization and functions of glycolipid-enriched microdomains in phagocytes. *Biochimica et Biophysica Acta*.

[91] Yuki N. (2012). Guillain-Barré syndrome and anti-ganglioside antibodies: a clinician-scientist's journey. *Proceedings of the Japan Academy—Series B: Physical and Biological Sciences*.

[92] Diamond B., Honig G., Mader S., Brimberg L., Volpe B. T. (2013). Brain-reactive antibodies and disease. *Annual Review of Immunology*.

[93] Chavada G., Willison H. J. (2012). Autoantibodies in immune-mediated neuropathies. *Current Opinion in Neurology*.

